# Cardiac Dysfunction in a Mouse Vascular Dementia Model of Bilateral Common Carotid Artery Stenosis

**DOI:** 10.3389/fcvm.2021.681572

**Published:** 2021-06-11

**Authors:** Lulu An, Michael Chopp, Alex Zacharek, Yi Shen, Zhili Chen, Yu Qian, Wei Li, Julie Landschoot-Ward, Zhongwu Liu, Poornima Venkat

**Affiliations:** ^1^Department of Neurology, Henry Ford Hospital, Detroit, MI, United States; ^2^Department of Physics, Oakland University, Rochester, MI, United States

**Keywords:** bilateral common carotid artery stenosis, brain–heart interaction, cardiac dysfunction, inflammation, oxidative stress

## Abstract

**Background:** Cardiac function is associated with cognitive function. Previously, we found that stroke and traumatic brain injury evoke cardiac dysfunction in mice. In this study, we investigate whether bilateral common carotid artery stenosis (BCAS), a model that induces vascular dementia (VaD) in mice, induces cardiac dysfunction.

**Methods:** Late-adult (6–8 months) C57BL/6J mice were subjected to sham surgery (*n* = 6) or BCAS (*n* = 8). BCAS was performed by applying microcoils (0.16 mm internal diameter) around both common carotid arteries. Cerebral blood flow and cognitive function tests were performed 21–28 days post-BCAS. Echocardiography was conducted in conscious mice 29 days after BCAS. Mice were sacrificed 30 days after BCAS. Heart tissues were isolated for immunohistochemical evaluation and real-time PCR assay.

**Results:** Compared to sham mice, BCAS in mice significantly induced cerebral hypoperfusion and cognitive dysfunction, increased cardiac hypertrophy, as indicated by the increased heart weight and the ratio of heart weight/body weight, and induced cardiac dysfunction and left ventricular (LV) enlargement, indicated by a decreased LV ejection fraction (LVEF) and LV fractional shortening (LVFS), increased LV dimension (LVD), and increased LV mass. Cognitive deficits significantly correlated with cardiac deficits. BCAS mice also exhibited significantly increased cardiac fibrosis, increased oxidative stress, as indicated by 4-hydroxynonenal and NADPH oxidase-2, increased leukocyte and macrophage infiltration into the heart, and increased cardiac interleukin-6 and thrombin gene expression.

**Conclusions:** BCAS in mice without primary cardiac disease provokes cardiac dysfunction, which, in part, may be mediated by increased inflammation and oxidative stress.

## Introduction

Vascular dementia (VaD) is one of the most common causes of dementia, responsible for ~15% of the cases after Alzheimer's disease (1). VaD has emerged as a predominant health problem worldwide in the elderly, with great impact on families and national economies, and there is no effective treatment (2). VaD is induced by a decrease in cerebral blood flow (CBF), which triggers inflammatory responses and oxidative stress, provoking lesions in the hippocampus, white matter, and basal ganglia (3). From a patient cohort study, a low cognitive score is associated with an increased incidence of cardiac vascular events (4). In addition, the presence of cardiovascular disease is associated with an increased risk of late-life cognitive impairment and dementia (5, 6).

Although there are extensive investigations on how cardiac dysfunction drives cognitive deficits (7), what has not been investigated is the converse, whether primary cerebrovascular events that result in cerebral hypoperfusion affect cardiovascular function. Our previous studies have found that brain damage, such as stroke and traumatic brain injury (TBI), induces cardiac deficit in mice even in the absence of primary cardiac diseases (8, 9). Here, we investigate whether bilateral common carotid artery stenosis (BCAS), which induces VaD in late-adult mice, induces cardiac deficits in non-primary cardiac disease mice. BCAS-induced pathologies parallel the underlying pathophysiological alterations evoking dementia found in patients with VaD (10–16). Therefore, the BCAS model was employed in this study. We propose that primary cerebral hypoperfusion which drives VaD provokes cardiac deficits.

## Materials and Methods

All experiments were conducted in accordance with the standards and procedures of the American Council on Animal Care and with the approval of the Institutional Animal Care and Use Committee of Henry Ford Health System. This manuscript was prepared in accordance with ARRIVE guidelines.

### BCAS Model and Experimental Procedure

Male (6–8 months old) C57BL/6J wild-type (WT) mice (weight = 28–32 g, *n* = 8) were subjected to BCAS, as previously described (10). Mice were anesthetized with 4% isoflurane in a chamber for pre-anesthesia and spontaneously respired with 2% isoflurane in a 2:1 N_2_O/O_2_ mixture using a facemask connected and regulated with a modified Fluotec 3 vaporizer (Fraser Harlake, Orchard Park, NY, USA). Both common carotid arteries (CCA) were exposed by a middle cervical incision. A microcoil (internal = 0.16 mm, spring: 5, material SUS304; RuikeBiotech Company, Xi'an, China) was applied by rotating it around the right CCA; after an interval of 1 h, a second microcoil was applied around the left CCA. The coils were permanently left in place. The early mortality in the BCAS group is 25% (two of eight mice). Sham mice (*n* = 6) were subjected to the same surgical procedures without the application of microcoils. Mice were sacrificed 30 days after BCAS for immunohistochemical staining and real-time PCR analysis. Several differences exist between employing 0.16- and 0.18-mm diameter coils. In a previous study, mortality with 0.16-mm microcoils was 75% and that with 0.18-mm microcoils was 15% at 14 days after BCAS (10). CBF recovered to ~70% of the baseline value in mice with 0.16-mm microcoils and remained significantly reduced compared to the control mice, whereas it recovered to ~80% and no significant difference was detected compared to the control group with the 0.18-mm microcoils 30 days after BCAS (12, 17). In the 0.18-mm microcoil model, there were no infarctions or hemorrhage in gray matter regions at 30 days. Animals with 0.16-mm microcoils exhibited focal infarction histological changes (10). Thus, using 0.16-mm microcoils induces more severe brain damage and chronic cerebral hypoperfusion. To reduce the early mortality, we employed an interval of 60 min between applications of microcoils.

### Cerebral Blood Flow Test

Regional CBF (rCBF) was measured in anesthetized mice 14 days after BCAS using laser Doppler flowmetry (LDF; PeriFlux PF4, Perimed AB, Datavagen, Sweden), as described previously (18, 19). Using a midline scalp incision, the skull was exposed and non-contact cortical rCBF was continuously recorded as perfusion units. The probe was placed in a fixed position of bregma and midline intersection while maintaining consistent acquisition parameters and region of interest for all mice at all time points of measurement.

### Cognitive Function Tests

Three cognitive function tests, including the Morris water maze (MWM) test (20), three-chamber sociability test (21), and the odor test (22), were performed following previously described methods 21–28 days after BCAS by an investigator blinded to the experimental groups. The modified neurological severity score (mNSS) was used for evaluating sensorimotor function.

#### Morris Water Maze

The MWM test, which spans 5 days and consists of four 90-s trials per day, is used to evaluate spatial and visual learning and memory. It relies on the navigation of mice in an open swimming pool to locate a submerged escape platform. The following parameters were recorded for each trial: escape latency, i.e., the time taken by the mice to reach the hidden platform, swim speed, and total swim distance. Data were collected using the ANY-Maze software, and the average over four trials for each day was calculated.

#### Three-Chamber Sociability Test

This test evaluates sociability and preference for social novelty. The testing apparatus consists of three interconnected chambers and the testing regimen comprises three phases: a habituation phase, a sociability task, and a preference for social novelty test. For the sociability task, the test mouse is given the option to interact with a stranger (S1) mouse housed within a small wire cage or an empty wire cage placed in the right and left compartments of the apparatus. For the social novelty task, the test mouse has the option to interact with the stranger 1 (S1) or a stranger 2 (S2) mouse, housed in wired cages in the right and left compartments of the apparatus. The times spent interacting with S1 vs. empty and S1 vs. S2 are recorded for the task.

#### Odor Test

The odor test evaluates olfactory learning and memory based on the animal's preference for new smells. The test spans 3 days, during which the animals are individually housed. The first day comprises habituation, during which four wooden beads (http://www.craftworks.com) were placed in the cages to serve as familiar odor (F). Day 2 comprises three trials, each spanning 1 min, during which the animals are familiarized to a donor smell (N1). Mice are freely allowed to explore three familiar beads (F) and one novel odor bead (N1) placed in the center of the cage. The position of N1 bead was randomly varied for each trial to minimize spatial cues, and an inter-trial interval of 1 mi void of any beads was used to minimize olfactory adaptation. Following a 24-h retention delay, mice are subject to a test phase spanning 1 min, during which the mice freely explore four beads (N1, N2, and two F beads) placed in the center of the cage. The time spent exploring (sniffing, licking, or biting) each bead was recorded during the test phase and a discrimination index calculated as the ratio between the time spent exploring N2 to the total exploration time.

#### Modified Neurological Severity Score

The mNSS includes a composite of motor, sensory, reflex, and balance tests, as previously described (23). On a scale of 0–18, 0 indicates the absence of neurological deficits, while higher scores indicate severe neurological injury and function.

### Echocardiography

Cardiac function was evaluated in conscious mice 29 days after BCAS. Transthoracic echocardiography measurements were obtained using an Acuson C516 (Siemens) machine equipped with a 15-MHz linear transducer (15L-8) (24, 25). Mice were trained for 3 days before performing echocardiography measurements to minimize bradycardia and the left hemithorax was shaved prior to testing. Each mouse was picked up by the nape of the neck, held firmly in the palm of one hand in the supine position, and a pre-warmed ultrasound transmission gel applied to the chest. M-mode images of the left ventricle (LV) from long-axis as well as short-axis views were recorded. All measurements were digitized by goal-directed, diagnostically driven software and three beats were averaged for each measurement. The left ventricular ejection fraction (LVEF) was measured using the formula: LVEF = [(LVAd – LVAs)/LVAs × 100], where LVAd is the LV diastolic area and LVAs is the LV systolic area. Relative wall thickness (RWT) was calculated as 2 × posterior wall thickness/LV end-diastolic diameter (26). All data were analyzed off-line at the end of the study with software resident on the ultrasound system, and all measurements were performed by an investigator who was blinded to the experimental groups.

### Immunostaining

Heart coronal sections (6 μm thick) were cut from paraffin-embedded blocks. Five sections from each heart, with each slide containing four fields of view, were imaged. Picrosirius Red (PSR, staining collagen I/III and muscle fibers as a fibrosis marker, 1:1,000; Sigma, St. Louis, MO, USA) staining was employed to measure perivascular fibrosis and interstitial collagen fraction (ICF). To study the immune response and oxidative stress after BCAS in the heart, antibody against CD45 (a marker for leukocyte, 1:250; Abcam, Cambridge, MA, USA), IBA1 (a marker for monocytes/macrophages, 1:1,000; Wako, Mountain View, CA, USA,), CD86 (M1 macrophage marker, 1:100; Abcam, Cambridge, MA, USA), 4-hydroxynonenal (4HNE, 1:1,000; Millipore Sigma, St. Louis, MO), and NOX-2 (NADPH oxidase 2, 1:400; BD, Franklin Lakes, NJ, USA) were employed. DAPI counterstain was used to stain nuclei in immunofluorescent staining. Negative controls consisted of similar procedures, but without the addition of the primary antibody.

For immunostaining measurements, positive cell numbers were counted for each field of view or the positive areas were measured using the densitometry function (MCID image analysis system) with a density threshold above the unstained set uniformly for all groups. Data from five sections, and four regions within each section, were averaged to obtain a single value for each animal and presented as the number of positive cells per square millimeter or the percentage of positive area.

### Polymerase Chain Reaction

To test the expressions of inflammatory cytokines, total heart RNA was isolated and utilized to perform quantitative PCR following standard protocol (27). The samples were tested by an investigator blinded to the experimental groups. Relative gene expression was analyzed using the 2^−ΔΔCt^ method.

GAPDH: Forward: GCCAAGGCTGTGGGCAAGGT
Reverse: TCTCCAGGCGGCACGTCAGA
Interleukin-6 (IL-6): Forward: TGATGCACTTGCAGAAAACA
Reverse: ACCAGAGGAAATTTTCAATAGGC
Thrombin: Forward: AGGACAATCTGTCACCTCCACT
Reverse: GGTCGAAGTCTTGGTACTTGCT.

### Statistical Analysis

Two-way analysis of variance (ANOVA) was utilized for the MWM outcome. Unpaired two-tailed Student's *t*-test was used to test significance of the two groups with GraphPad Prism 8 (Graph Pad Software Inc., San Diego, CA, USA). The relationship between the cognitive functional outcome and LVEF was analyzed using Pearson's correlation coefficients. ^*^P < 0.05 was considered statistically significant. Data in all figures are presented as the mean ± SEM.

## Results

### BCAS Induces Chronic Hypoperfusion and Cognitive Deficit

**Figure 1:**
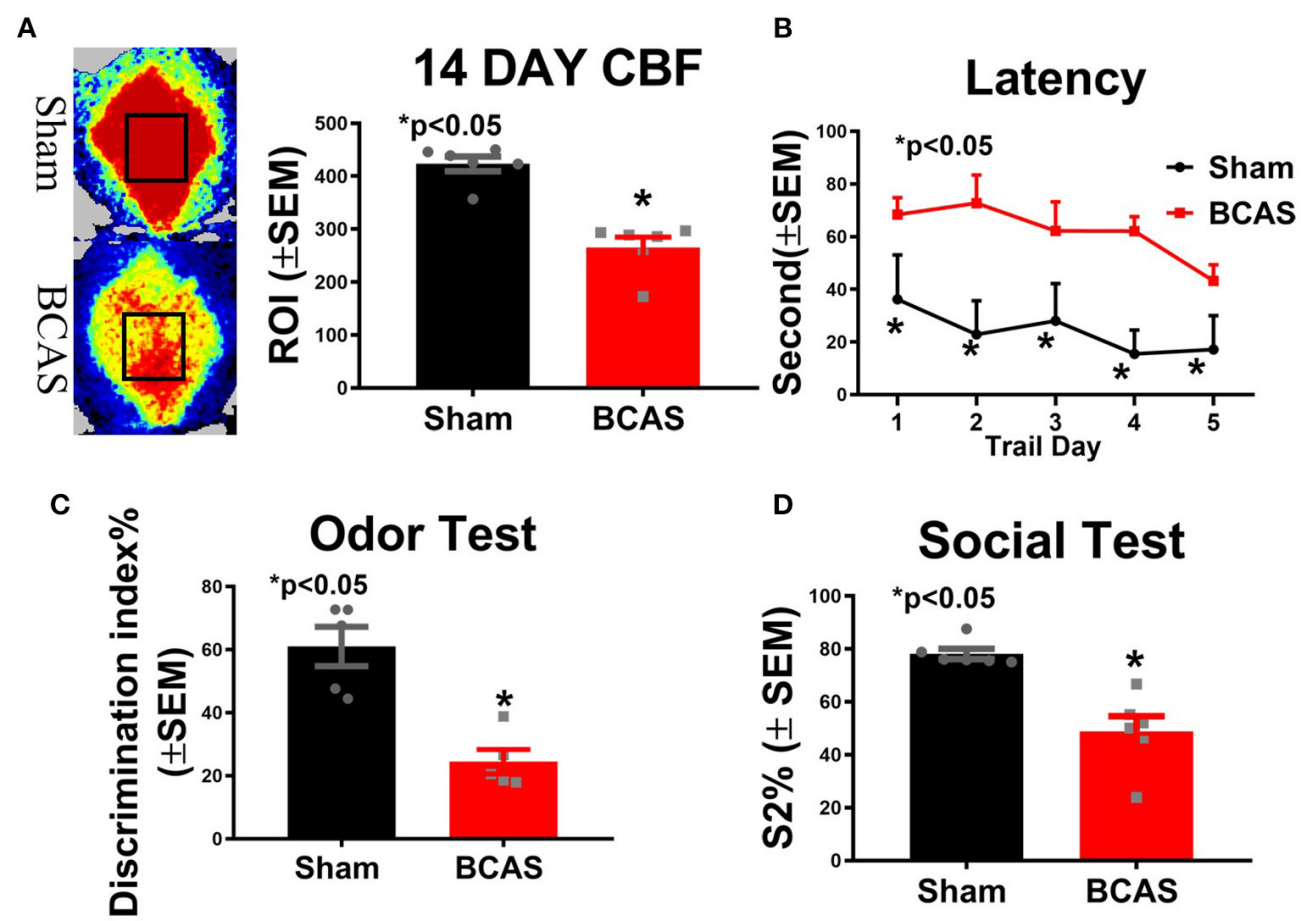
Bilateral common carotid artery stenosis (BCAS) significantly induces hypoperfusion and cognitive dysfunction compared to sham mice. **(A)** Cerebral blood flow (CBF) measurement 14 days after BCAS. **(B)** Latency of the Morris water maze (MWM). **(C)** Odor test. **(D)** Social test. *n* = 6/group. *p < 0.05.

To test whether BCAS induces chronic cerebral hypoperfusion, CBF was measured at day 14 after BCAS (Figure 1). Figure 1A shows a significantly decreased CBF after BCAS compared to the sham group. To investigate whether BCAS induces cognitive deficit, three function tests were employed. We found that BCAS mice exhibit a significantly increased escape latency on all 5 days of the MWM test compared to sham (Figure 1B). BCAS significantly decreases the discrimination index in the odor test and the social test, indicating significant memory impairment compared to sham mice, as shown in Figures 1C,D. Our data indicate that BCAS significantly induces chronic cerebral hypoperfusion and leads to cognitive impairment. On a scale of 0–18, where 0 indicates a lack of neurological deficits and 18 indicates maximum and severe neurological deficits, an mNSS score <6 is generally considered to indicate mild neurological deficits with small to no ischemic infarct. Compared to the sham control group, VaD mice exhibited mild neurological deficits on day 21 after BCAS (control = 0 ± 0; BCAS = 4.6 ± 1.03). Also, the mean swim speed of BCAS mice was significantly reduced on days 1 and 4 of the MWM test compared to sham mice (Supplementary Figure 1A). Therefore, we cannot rule out that motor deficits may contribute to some of the differences observed in the MWM test.

### BCAS Induces Cardiac Hypertrophy and Dysfunction Compared to Sham Mice

**Figure 2:**
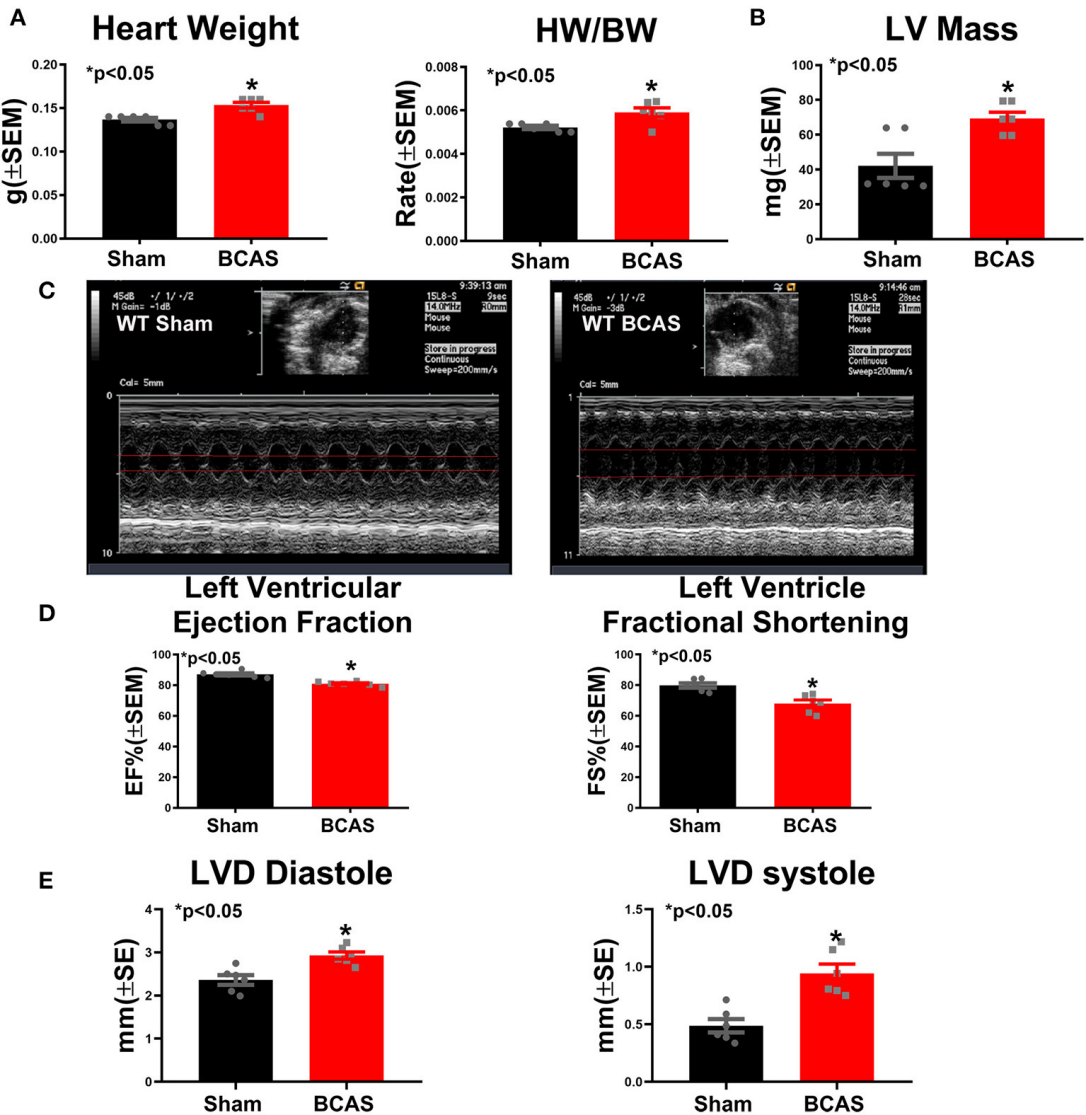
Bilateral common carotid artery stenosis (BCAS) induces cardiac hypertrophy and cardiac dysfunction compared to sham mice. **(A)** Heart weight (HW) and ratio of HW to body weight (BW). **(B)** Left ventricular (LV) mass. **(C)** Echocardiography figures of sham and BCAS mice. **(D)** Left ventricular ejection fraction (LVEF) and left ventricular fractional shortening (LVFS) measurements. **(E)** Left ventricular diameter (LVD) at diastole and systole. *n* = 6/group. *p < 0.05.

To test whether BCAS affects cardiac function, heart weight and echocardiography measurements were performed in conscious mice (Figure 2). The heart rate and systolic function in conscious mice are typically much higher than those seen in anesthetized mice (28, 29). Figure 2A shows that BCAS significantly increases the heart weight and the ratio of heart weight/body weight (HW/BW). Figures 2B–E and Supplementary Figures 1B,C show that BCAS mice exhibit significantly increased LV mass, reduced LVEF and left ventricular fractional shortening (LVFS), and increased left ventricular dimension (LVD) without any change in the heart rate compared to sham control mice as measured by echocardiography. BCAS mice exhibited a declining trend in RWT (*p* = 0.09). Our data indicate that BCAS results in cardiac systolic dysfunction and eccentric hypertrophy in mice even in the absence of primary cardiac disease.

### Cognitive Dysfunction Significantly Correlates to Cardiac Dysfunction

**Figure 3:**
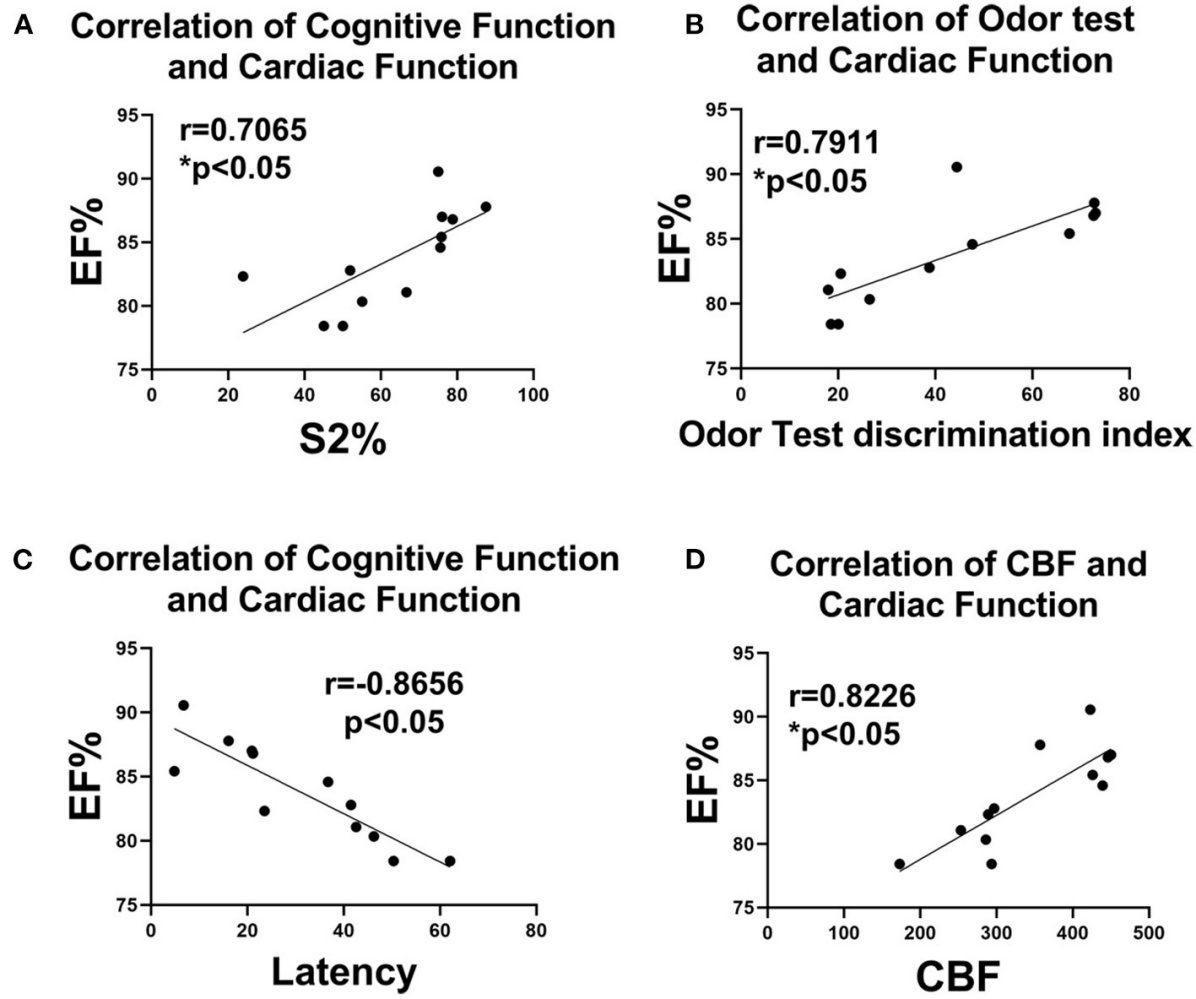
Correlation analysis of cognitive and cardiac functions. Correlation analysis of **(A)** social test S2% discrimination and EF%, **(B)** odor test discrimination and EF%, **(C)** latency and EF%, and **(D)** CBF and EF%. *n* = 6/group. *p < 0.05.

Pearson's correlation test (Figure 3) was used to evaluate the correlations between cognitive dysfunction and cardiac dysfunction, and we found that the sociability test S2% discrimination index, odor test discrimination, CBF, and MWM latency were significantly correlated with LVEF, as shown in Figures 3A–D. LVEF is a primary index of cardiac function (30). Our data indicate that cardiac dysfunction is related to cognitive dysfunction.

### BCAS Induces Cardiac Fibrosis and Oxidative Stress Compared to Sham Mice

**Figure 4:**
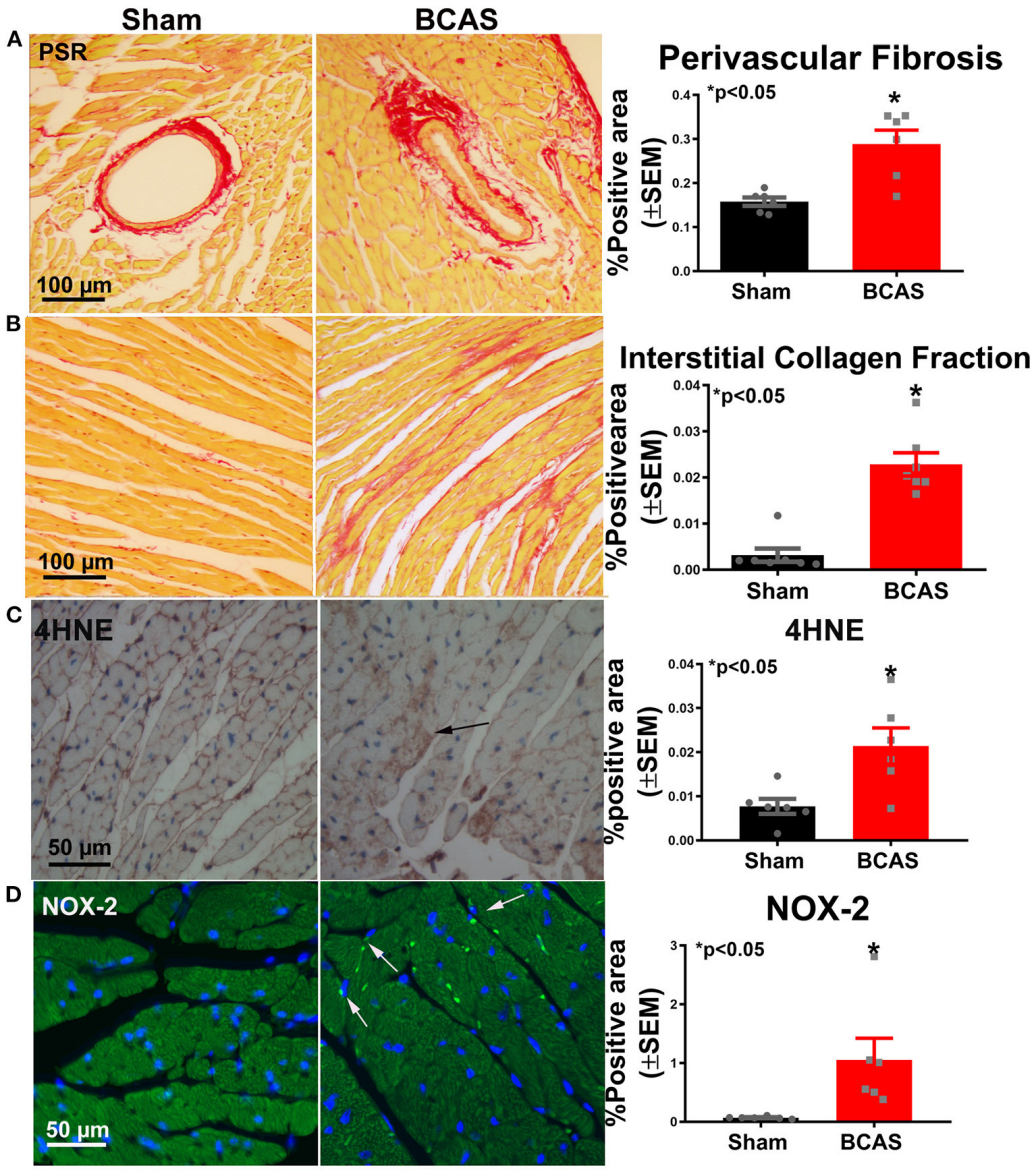
Bilateral common carotid artery stenosis (BCAS) significantly increases cardiac fibrosis and oxidative stress compared to sham mice. **(A)** Picrosirius Red (PSR) staining and perivascular fibrosis measurement. **(B)** PSR staining and interstitial fibrosis measurement. **(C)** 4-Hydroxynonenal (4HNE) staining and quantitative data. **(D)** NADPH oxidase 2 (NOX-2) staining and quantitative data. *n* = 6/group. *p < 0.05.

PSR staining was performed (Figure 4) to investigate whether BCAS induces cardiac fibrosis. BCAS mice exhibited significantly increased interstitial and perivascular fibrosis compared to sham mice, as shown in Figures 4A,B. Our data indicate that BCAS induces cardiac fibrosis. Immunostaining was employed to assess oxidative stress in the heart. Figures 4C,D indicate that BCAS significantly increases 4HNE and NOX-2 in the BCAS group compared to the sham group. Our data indicate that BCAS increases oxidative stress in heart.

### BCAS Significantly Increases Cardiac Inflammatory Response

**Figure 5:**
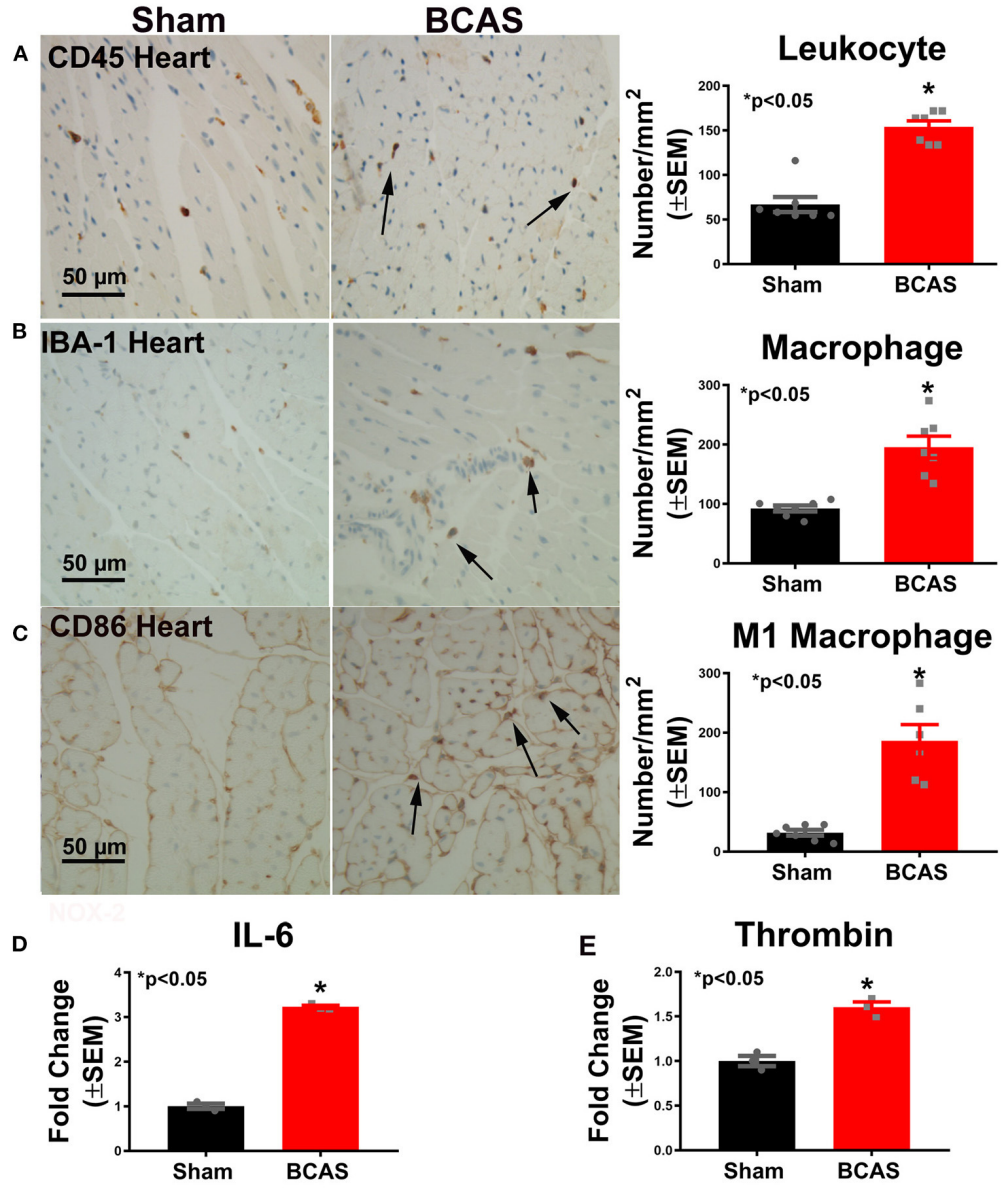
Bilateral common carotid artery stenosis (BCAS) significantly increases inflammatory cell and inflammatory factor gene expression in the heart compared to sham mice. **(A)** CD45 immunostaining in the heart tissue and quantitative data. **(B)** IBA-1 immunostaining and quantitative data. **(C)** M1 macrophage immunostaining and quantitative data. **(D)** IL-6 gene expression. **(E)** Thrombin gene expression. *n* = 6/group. *p < 0.05.

To determine whether BCAS induces inflammatory cell infiltration, we employed immunostaining (Figure 5). Figure 5A shows that BCAS significantly increased leukocyte infiltration compared to sham mice. Macrophage infiltration increased in BCAS mice compared to sham mice, as shown in Figure 5B. To determine the macrophage phenotype, we employed CD86 immunostaining and found that M1 macrophage increased in the BCAS group compared to the sham group (Figure 5C).

To investigate possible mechanisms by which BCAS induces cardiac dysfunction, real-time PCR was employed. Figures 5D,E show that the expressions of the inflammatory cytokines IL-6 and thrombin significantly increased in the heart of mice subjected to BCAS compared to sham mice, respectively. Our data indicate that BCAS induces increased inflammatory factor expression in the heart.

## Discussion

In this study, we used a BCAS model to induce VaD. We found that BCAS not only significantly induces chronic hypoperfusion, and leads to cognitive function impairment, but also increases cardiac hypertrophy and cardiac dysfunction. BCAS-induced cognitive deficit is significantly correlated with cardiac deficit. BCAS leads to cardiac fibrosis, increases leukocyte and M1 macrophage infiltration, and induces oxidative stress and inflammatory factor expression of IL-6 and thrombin in the heart. These data suggest that inflammation and oxidative stress may contribute to the cardiac dysfunction in BCAS mice.

### BCAS Induces Hypoperfusion and Cognitive Deficits

The BCAS model induces chronic cerebral hypoperfusion and serves as a powerful tool for the investigation of VaD (17). In our study, we found that BCAS leads to a chronic decrease in CBF in mice, and these mice develop cognitive impairment. Thus, this model of BCAS appears as a reasonable model of VaD and reflects its underlying mechanisms. We found that mice subjected to BCAS exhibit significant cognitive impairment, as indicated by the reduced discrimination index in the novel odor recognition test and the reduced preference for social novelty in the three-chamber social interaction test. There have been mixed reports regarding spatial memory impairment evaluated using the MWM test following BCAS. While some studies report selective impairment of spatial and working memory after BCAS without changes in the escape latency or swim speed in MWM between sham and BCAS mice (11, 31), other studies have reported significantly increased escape latency in the MWM test when evaluated at 4, 6, or 8 weeks post-BCAS in young mice compared to sham control mice (32–35). In our study, BCAS mice exhibited significantly increased escape latency on all 5 days of testing compared to sham control mice. In addition, we found that BCAS mice exhibit mild neurological deficits in the mNSS test and have reduced swim speed in the MWM test. Therefore, we cannot rule out that motor deficits may contribute to some of the cognitive differences observed in the MWM test.

### BCAS Induces Cardiac Dysfunction

Neurocardiology is an emerging field that elucidates the effect of cerebral lesion on the heart (36). Dementia patients with a prior history of heart disease are more likely to have structural and functional cardiac abnormalities compared with control normal subjects (36). Heart failure is correlated with dementia in the elderly, and most clinical patients with heart failure had dementia, but with dementia onset prior to evident heart failure (HF), which suggests that dementia may trigger subclinical onset of heart failure (37). Reduced CBF triggered by cardiac dysfunction further exacerbates VaD and Alzheimer's disease (AD) pathology (38).

In our previous studies, we also observed cardiac dysfunction and cardiac fibrosis at 28 days after intracerebral hemorrhage (39), 30 days after ischemic stroke (25, 40), as well as 30 days after traumatic brain injury (9). In the present study, our data show that BCAS induces significant cardiac eccentric hypertrophy, cardiac dysfunction, and cardiac fibrosis compared to sham mice. Additionally, a significant correlation between cardiac dysfunction and cognitive dysfunction is shown *via* the Pearson's correlation test. To our knowledge, we are the first to demonstrate that BCAS-induced VaD leads to cardiac dysfunction in mice without primary heart disease.

### BCAS Induces Cardiac Fibrosis

Many mechanisms may regulate brain–heart interaction after brain damage. Inflammatory cell infiltration into the heart and increased inflammatory factor expression may lead to progressive cardiac functional deficits after BCAS. Inflammation may be a mediator of cardiac fibrosis and heart failure (41). Macrophages play critical roles in the initiation and progression of fibrotic responses, which are dependent on microenvironmental factors (42). Macrophages may differentiate into myofibroblasts and secrete cytokines and growth factors to increase fibrosis (43). Homeostasis of the cardiac fibroblast results in the disruption of myocardial excitation–contraction coupling in both systole and diastole, and disruption of this homeostasis leads to a far-reaching impairment of systolic and diastolic function (43). Activated macrophage infiltration into the heart tissue releases fibrogenic cytokines that promote fibrosis and remodeling, eventually leading to cardiac dysfunction (44). In our study, we found that macrophages are increased in the heart of BCAS mice. BCAS may evoke an increase in cardiac macrophages, which release fibrogenic cytokines, leading to the deposition of collagen and subsequent cardiac dysfunction (45).

### BCAS Increases Cardiac Inflammation

Previous studies found that immune response plays a vital role in regulating brain–heart interaction after stroke, TBI, subarachnoid hemorrhage (SAH), and intracerebral hemorrhage (ICH) (8, 9, 44, 46). Inhibition of the immune response after stroke, TBI, SAH, and ICH by splenectomy significantly reduces inflammatory cell infiltration into the heart and decreases the inflammatory factor and oxidative stress response in the heart, which may mediate the splenectomy-reduced cardiac deficit after stroke and TBI (8, 9, 44, 46). The phenotype and function of macrophages are regulated by the surrounding microenvironment and can be classified into two types, with the classically activated or M1 macrophage regarded as pro-inflammatory and the M2 macrophage as anti-inflammatory (47). M1 macrophages induce tissue damage and initiate inflammatory responses that result in cardiac long-term pathological remodeling (48–50). M1 macrophages produce many cytokines, including IL-6 (51). IL-6 is a pleiotropic cytokine, and in the acute phase of ischemia–reperfusion injury and myocardial infarction in a rat model, it acts as a protective cytokine to host injury while chronically becomes pathogenic to the host, leading to chronic inflammation and cardiac fibrosis (52). Meta-analysis of human studies has shown that long-term elevation of IL-6 more than doubles a person's lifetime risk of coronary heart disease (53). Increased expression and release of the inflammatory cytokine IL-6 are observed in HF patients (54). IL-6 promotes the infiltration and activation of mononuclear leukocytes (55) and activates the coagulation pathway and vascular endothelial cells, but inhibits myocardial function (56). In the present study, we found that M1 macrophage infiltration into the heart and the expression of IL-6 in the heart were elevated after BCAS, which may contribute to BCAS-induced cardiac dysfunction.

### BCAS Induces Oxidative Stress Response in the Heart

Growing evidence highlights oxidative stress as an important mechanism in cardiac deficit (57). Excessive oxidative stress response impairs the DNA/RNA synthesis and causes cellular damage, while fibroblasts have been well-documented to utilize reactive oxygen species (ROS) in differentiation to secrete collagen, which contributes to cardiac fibrosis (58). 4HNE is a reactive lipid mediator generated from lipid peroxidation, which is a biomarker of oxidative stress (59). The degree of lipid peroxidation is correlated to the severity of cardiovascular disease (CVD) in patients (60), and 4HNE adducts to mitochondrial NADP^+^-isocitrate dehydrogenase and was shown to enhance cardiac hypertrophy in a spontaneously hypertensive rat model (61). NADPH oxidase is an important ROS source in cardiac cells and modulates several key processes underlying the myocardial response to injury (62). NOX-2 is the main isoform expressed in the myocardium (63). ROS, especially from NOX-2, make a vital contribution to the development of cardiac dysfunction and remodeling (62, 64). NOX-2 knockout mice exhibit cardiac protective effects associated with the decrease of oxidative stress and neutrophil invasion in an ischemia–reperfusion model (65). NOX-2 also drives macrophages turning into M1 macrophages (66, 67), which further aggravates the damage. In our study, we found that BCAS significantly increases 4HNE and NOX-2 expression in the heart tissue compared to sham mice. Collectively, oxidative stress may contribute to cardiac deficits after BCAS.

### BCAS Increases Thrombin Expression in the Heart

Thrombin, a potent platelet activator, is the central protease of the coagulation cascade, and it participates in left atrial remodeling (68), cardiac hypertrophy, and heart failure (69). Thrombin signals act *via* protease-activated receptor (PAR)1 and PAR4 on human platelets, which have been implicated in the generation of ROS (70). In addition, subjects with heart failure are at higher risk of developing thrombosis, and thrombin generation is accelerated and amplified in heart failure (71). *In vitro*, murine macrophages can be stimulated to polarize into M1 by thrombin *via* PAR1 (72). Moreover, thrombin induces IL-6 production in fibroblasts (73), which exacerbates tissue injury. Thrombin overload induces cardiac fibrosis and dysfunction in mice (74). Thrombin is highly correlated to NOX-2 since NOX-2 is the key target to activate oxidative stress-mediated platelet activation and thrombosis (75). Inhibitors of NOX-2 block thrombin-induced platelet secretion, aggregation, and ROS generation (76, 77). In this study, we found that BCAS increases not only M1 macrophage, IL6, and oxidant stress but also thrombin expression in the heart. Thus, increased thrombin may also contribute to BCAS-induced cardiac deficit.

## Limitations

In this study, we demonstrate that BCAS induces cardiac deficit and increases cardiac inflammation, oxidative stress, and thrombin expression. However, the mechanisms of BCAS-induced cardiac deficit have not been definitively demonstrated and warrant further investigation. There are many mechanisms involved in brain–heart interaction. The HPA axis consisting of the hypothalamus, pituitary, and adrenal glands is an important mediator of brain–heart interaction (77). The catecholamine released may contribute to cardiac deficits after VaD. Sympathetic response and elevated systemic catecholamine levels have been associated with cardiac dysfunction (78). Sympathetic nerve terminals can release catecholamines directly into the heart, while the adrenal medulla releases catecholamines into the blood stream and thereby provoke heart deficit (79). Takotsubo syndrome (TKS) is an acute cardiomyopathy which is driven by stressful emotional or physical conditions (80). Catecholamine-induced myocardial stunning is one of the suggested mechanisms of TKS (81), which may also be related to BCAS-induced cardiac deficit. The renin–angiotensin–aldosterone system (RAAS), also referred to as the angiotensin (Ang)-converting enzyme/Ang II/Ang type 1 receptor axis, is altered following cerebrovascular injuries such as stroke and BCAS-induced hypoperfusion and is known to induce injury in various organs, affecting cardiac function, renal function, as well as cognitive function (32, 82, 83). RAAS blockers are effective in reducing left ventricular hypertrophy, fibrosis, diastolic dysfunction, and associated cardiac dysfunction (84). Specifically, in BCAS, direct activation of angiotensin type 2 receptor was found to increase CBF and reduce inflammation, thereby improving cognitive function in mice (32). RAAS plays an important role in CBF regulatory mechanisms (85, 86). Reduced CBF can also increase the secretion of aldosterone from the adrenals, which in turn can contribute to cardiac remodeling following BCAS. The role of the RAAS in BCAS-induced cardiac dysfunction remains to be determined, and further studies are warranted. The nervous system and the immune system affect each other *via* sympatho-adrenergic pathways, suggested by dopaminergic receptor expression on immune cells (87). Brain-derived microvesicles (BDMVs) and microRNA mediate intercellular and inter-organ communication (36). BDMVs released from injured brain induce systemic coagulopathy, which may induce cardiac deficits (36, 88). Future studies are warranted to investigate the mechanisms and test interventions to improve neurocognitive as well as cardiac function in VaD. In addition, in this study, we only employ adult male mice; however, we cannot ignore the effects of age and gender on dementia. With aging and gender differences, the mechanisms of BCAS-induced cardiac deficits warrant further investigations.

## Conclusions

BCAS in male mice induces cognitive and heart deficits. The cardiac dysfunction is significantly correlated with cognitive deficit in BCAS mice. BCAS also increases the inflammatory and oxidative stress response in the heart in non-primary cardiac disease mice. Increasing inflammation and oxidant stress in the heart may contribute to BCAS-induced cardiac deficit.

## Data Availability Statement

The original contributions presented in the study are included in the article/Supplementary Material, further inquiries can be directed to the corresponding author/s.

## Ethics Statement

The animal study was reviewed and approved by Institutional Animal Care and Use Committee of Henry Ford Health System.

## Author Contributions

LA, AZ, YS, ZC, YQ, WL, and JL-W: formal analysis and investigation. LA and PV: manuscript preparation. PV, ZL, and MC: supervision and revision. All authors contributed to the article and approved the submitted version.

## Conflict of Interest

The authors declare that the research was conducted in the absence of any commercial or financial relationships that could be construed as a potential conflict of interest.
