# Supplementary material for: Cardiac Dysfunction in a Mouse Vascular Dementia Model of Bilateral Common Carotid Artery Stenosis
Source: Front Cardiovasc Med. 2021 Jun 11;8:681572. doi: 10.3389/fcvm.2021.681572 (PMC8225957; doi:10.3389/fcvm.2021.681572)
Supplement: Supplementary file 1 [file Data_Sheet_1.pdf]

Supplementary Data

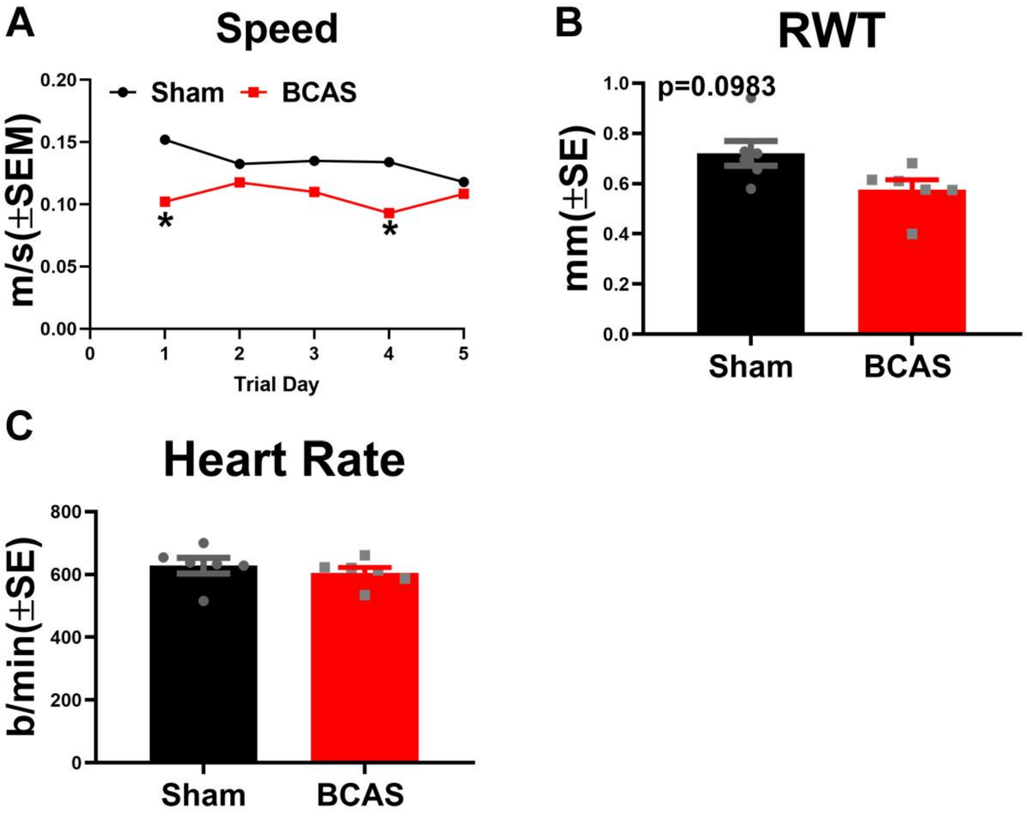

Supplementary Figure 1: (A) Swim speed in Morris Water Maze test and (B) Relative Wall Thickness (RWT) and (C) heart rate of BCAS and sham mice measured by echocardiography in consious mice. n=6/group, *p<0.5.
